# Honey Bees Modulate Their Olfactory Learning in the Presence of Hornet Predators and Alarm Component

**DOI:** 10.1371/journal.pone.0150399

**Published:** 2016-02-26

**Authors:** Zhengwei Wang, Yufeng Qu, Shihao Dong, Ping Wen, Jianjun Li, Ken Tan, Randolf Menzel

**Affiliations:** 1 Key Laboratory of Tropical Forest Ecology, Xishuangbanna Tropical Botanical Garden, Chinese Academy of Sciences, Kunming, China; 2 Eastern Bee Research Institute, Yunnan Agricultural University, Kunming, China; 3 Institute of Biology and Neurobiology, Freie Universität Berlin, Berlin, Germany; Goethe University Frankfurt, GERMANY

## Abstract

In Southeast Asia the native honey bee species *Apis cerana* is often attacked by hornets (*Vespa velutina*), mainly in the period from April to November. During the co-evolution of these two species honey bees have developed several strategies to defend themselves such as learning the odors of hornets and releasing alarm components to inform other mates. However, so far little is known about whether and how honey bees modulate their olfactory learning in the presence of the hornet predator and alarm components of honey bee itself. In the present study, we test for associative olfactory learning of *A*. *cerana* in the presence of predator odors, the alarm pheromone component isopentyl acetate (IPA), or a floral odor (hexanal) as a control. The results show that bees can detect live hornet odors, that there is almost no association between the innately aversive hornet odor and the appetitive stimulus sucrose, and that IPA is less well associated with an appetitive stimulus when compared with a floral odor. In order to imitate natural conditions, e.g. when bees are foraging on flowers and a predator shows up, or alarm pheromone is released by a captured mate, we tested combinations of the hornet odor and floral odor, or IPA and floral odor. Both of these combinations led to reduced learning scores. This study aims to contribute to a better understanding of the prey-predator system between *A*. *cerana* and *V*. *velutina*.

## Introduction

The appetitive form of associative learning plays a major role in honey bee (*Apis mellifera*) foraging. Foragers learn to associate visual and olfactory stimuli with a food reward quickly and reliably, and form a long-lasting memory of this association [1,2]. Workers can also learn to respond to an odor stimulus in a laboratory setting. In what has become known as the proboscis extension response or PER paradigm [3,4] a harnessed bee learns to associate a sucrose reward with an odor stimulus offered immediately prior to the reward. Memory formation is evidenced when the harnessed bee extends its proboscis in response to the learned odor in anticipation of the sucrose reward. This form of classical conditioning has been successfully used to characterize multiple characteristics of associative learning [5–7] and also proved amenable in *A*. *cerana* [8] and *A*. *florea* [9]. The acquisition process, the dynamics of memory formation and the corresponding neural and molecular mechanisms of memory formation in the bee can be studied in great detail with this paradigm [7,10,11].

The odors applied in appetitive PER conditioning are generally floral-like, such as hexanal or nonanal [12]. However, training can also be carried out successfully with non-floral odors such as the bee´s Nasonov pheromone components geraniol and citral [13,14] and some pesticides [15–17]. For example, *A*. *mellifera* can be trained to respond with proboscis extension even when the learned odor is the sting pheromone, indicating that workers can override their innate response to sting pheromone and learn to associate this odor with a food reward [18,19]. This shows that even an aversively-laden odor like sting pheromone can still be learned in an appetitive context. Context dependence of the behavioral response to odors has also been demonstrated in ants [20]. We wondered if odors indicating predation risk, such as hornet odors and the main components of alarm pheromone of honey bees, can be overcome and converted to an appetitive stimulus by reward learning in eastern bees, and whether the hornet odor or the sting pheromone can alter learning of a floral odor.

The hornet *Vespa velutina* is endemic to Southeast Asia and preys on bees and other insects [21]. When under attack by a hornet, workers of *A*. *cerana* and *A*. *mellifera* recruit nest mates to aggregate at the nest entrances, shimmer and engage in heat balling if the hornet gets closer [22–27]. Since the establishment of this hornet species in Europe, attention has focused increasingly on the relationship between bees and hornets [28,29]. In recent years, we found that *A*. *cerana* bees detect *V*. *velutina* when the hornet hovers at the bee hive entrance. Guards will shimmer to repel the hornet attack and this shimmering behavior is innate to *A*. *cerana* [30,31]. As the hornets get closer, the risk of attack increases, more bees are recruited, and the shimmering strength increases [31]. Those results indicated that *A*. *cerana* may judge the predation-risk level, and adjust their defensive strategies accordingly.

Breed et al. [32] divided honey bee defensive responses into different sequences of events—perception, orientation, discrimination and identification of the predator, then recruitment of other nest mates, and finally attack. Alarm pheromones not only direct the recruited bees towards the predator, they also guide their attack. Isopentyl acetate (isoamyl acetate, or IPA) is one of the principal active alarm pheromone components in the genus *Apis* [33,34]. In the present study, we report investigations into learning of *A*. *cerana* under predation risk and ask whether: (1) worker bees can detect live hornet odors, (2) associate hornet odors and honey bee main alarm pheromone IPA with sucrose reward, (3) bees respond differently to predator odor and IPA, and (4) whether the predator odors modulate olfactory learning of flower odors.

## Methods

The experiments were performed from April to November, when eastern bees (*Apis cerana*) and hornets (*Vespa velutina*) coexist, in the experimental apiary of Eastern Bee Research Institute in Yunnan Agricultural University campus. Six bee colonies were housed in standard Langstroth hives and each colony comprised two frames of brood and two frames of honey and pollen (Which is a normal strength for an *A*. *cerana* colony).

To determine if *A*. *cerana* foragers could detect live hornet (*V*. *velutina*) odors, electroanatennography (EAG) was used to test the bees’ antennal responses. Six foragers were taken from each of three colonies. Each antenna of one bee was used to test the response to three control and three hornet odors, with a control and hornet inter-present sequence. A live hornet was trapped in a PTFE tube where it served as a hornet odor generator; the control group had no hornet in the tube. All EAG tests were conducted on sunny days only. We first carefully captured an *A*. *cerana* forager, chilled it briefly on ice, cut off its antennae, and placed each antenna into a glass body Ag/AgCl electrode filled with insect Ringer’s solution [35]. Each antenna was placed 1 cm away from the outlet of a PTFE tube (1 cm inner diameter, 15 cm long) that provided the test odor by combining a clean (500 mL active charcoal filtered) and wet (distilled water, 90% RH) 15 ml/s continuous air flow and a pre-filtered and wet 5 ml/s pulsed air flow (90% RH) with the test odor (hornet odor or control odor). For each stimulation, pulsed odor air flow was delivered into the odor pipette for 3 s, mixing into the continuous flow. To record the antennal responses, we used a custom stimulus controller, a modified EAG Combi Probe amplifier (Syntech, NL, but modified to increase sensitivity) outputting a signal into an HP34405A Digital Multi Meter (Agilent, USA) and BenchVue software (Keysight, USA) running on a PC.

### Conditioning experiment general setups

Returning nectar foragers were captured in small vials at the entrance of the hive on sunny days, pollen foragers were excluded from our collection. The bees were then transferred back to the laboratory and were chilled until motionless and then mounted in small plastic tubes leaving the bee head and proboscis flexible [8]. The bees were placed in an incubator (24°C, 65% RH) for five hours. Two hours before each experiment the bees were stimulated with sucrose solution (US) and then with an odor (CS). Bees that showed the PER to the odor prior to conditioning or failed to exhibit a PER in response to sucrose stimulation were discarded from the experiment.

In the test of whether honey bees modulate their olfactory learning in the presence of the hornet predator and the alarm pheromone components of honey bee itself. Live hornets were captured with a nylon trap net and placed individually in a plastic syringe (60 ml) which served as a hornet olfactory stimulus generator. Hexanal and nonanal (Hexanal, 98%, Sigma-Aldrich Co., St. Louis, USA; Nonanal, 98%, Sigma-Aldrich Co., St. Louis, USA) were used as floral control odors in PER conditioning. Two μl hexanal were placed on a strip of filter paper (5×5 mm) in a plastic syringe (20 ml). Two μl isopentyl acetate (IPA, 98%, Aladdin Reagent Database Inc. Shanghai, China), a classic component found in bee alarm pheromone, was placed on filter paper (5×5 mm) in a plastic syringe (20 ml), and used as a substitute for alarm pheromone. Three syringes were placed with their tips 1.5 cm from the bee’s head. One syringe contained only filter paper and delivered a constant airflow to reduce learning of the mechanosensory component of the stimulus. The other two syringes containing hexanal or the treatment odor (hornet odor, IPA or nonanal) were attached to a valve at the end of a Y-shaped silicon tube, and an air pump. The bees were placed in a continuous airflow (main airflow of 50 mls^-1^) which was switched between two syringes, one with the control odor (hexanal) and one with treatment odor (hornet odor, IPA or nonanal). The rewarding unconditioned stimulus (US) was 30% (m/v) scentless sugar syrup. An air stream exhaust system was formed by a 10 cm diameter tube that was placed 12 cm behind the bee.

### Experiment 1 Hornet odor and IPA conditioning

Olfactory conditioning of PER was performed according to Bitterman et al. [4] except that the bees were conditioned on the same day as they were captured. Hornet odor and IPA was used as a conditioned stimulus, respectively; and the floral odor hexanal was used as a control stimulus. During the PER conditioning, the conditioned stimulus was presented for 5 s, the unconditioned stimulus started 3 s after onset of the CS and lasted for 3 s. The US was delivered with a toothpick containing the sucrose solution. First the antennae were touched and then the proboscis. Each individual bee underwent six training trials. The interval between two trials was 10 min. One hour after the last training trial, all bees were tested with respect to their retention of the CS by presenting the CS only. Three groups of animals were run in parallel. In total, 118 worker bees were tested with hornet odor, 120 worker bees with IPA and 120 bees with hexanal.

### Experiment 2 Two-odor combination conditioning

In order to imitate natural conditions whereby a bee encounters a hornet predator when foraging on a flower or receives the alarm signal from its mates, the bees were first stimulated with hornet odor or IPA for 5 s and then with hexanal for another 5 s. From the third second of hexanal onwards the bees were rewarded with sucrose. A combination of nonanal (5s) and hexanal (5s) stimuli was applied to a control group. Six training trials were applied to each individual bee. The interval between the trials was 10 min. Each bee was tested for its response to the CS alone one hour after the last training trial. Each group of bees achieved a retention score expressed in the percentage of bees of each group responding to the conditioned odors. In total, 107 worker bees were tested with hornet-hexanal, 110 worker bees with IPA-hexanal and 110 bees with nonanal-hexanal.

### Statistics

To test the olfactory response of bees to live hornets, mean responses (peak amplitudes were used as the response to odors, Log transformed EAG responses) were to each of the three trials of control odors and hornet odors obtained from each bee antenna. We used univariate ANOVA with different odors and different bees from different colonies as a fixed factor to determine if the bee can discriminate hornet odor from the control odor.

Data were recorded as the proportion of bees exhibiting the PER in each of the experimental groups (retention score). These data were analyzed with a repeated measures ANOVA, using trials as the within-subject effect and different odor groups (hexanal, hornet odor and IPA) as the between-subject effect, respectively. We performed Mauchly’s sphericity test to ensure that the assumption of sphericity was not violated. Alternatively, we used the Greenhouse-Geiser degrees of freedom adjustment for sphericity. A post hoc test (least significant differences, LSD) was used to determine if there were significant differences between the predator odor group, alarm odor group and control group, respectively.

A one-way ANOVA was used to determine if there were any differences in retention scores among the hornet odor group, IPA group and hexanal group in hornet and IPA conditioning experiments. Paired t-tests were used to determine if there were differences in the retention scores between experienced odors and non-experienced odors in two odor combination experiments. The retention scores among these three groups (hornet-hexanal combination group, IPA-hexanal combination group and nonanal-hexanal group) were compared with a one-way ANOVA. All calculations were conducted with SPSS Statistics 19.0 (www.spss-china.com).

## Results

### Can live hornet odor be detected by bees?

**Fig 1:**
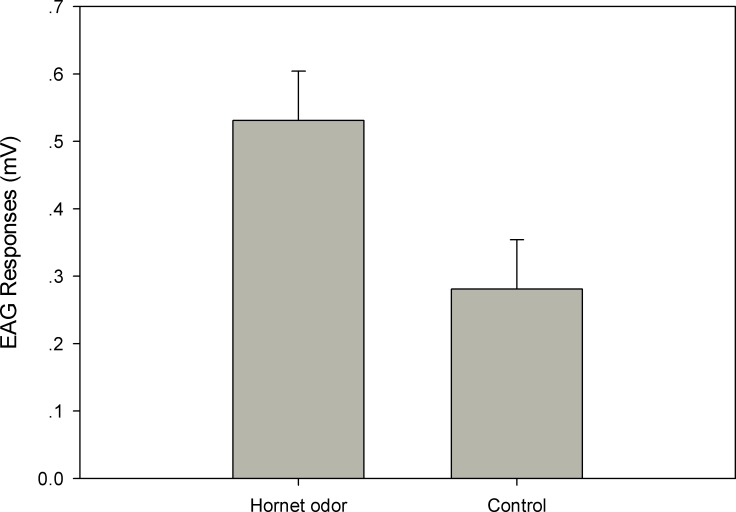
EAG responses of *A*.*cerana* to live hornet odor.

In total, 18 bees were tested with hornet odor, and 18 bees were tested with the control odor. The EAG responses showed that *A*. *cerana* can discriminate hornet odor from the control odor, which showed higher antennal responses to hornet odor (*F*_1,35_ = 5.82, *P* = 0.03). However, no difference was found between different bees from different colonies (colony effect: *P* = 0.06, different bees: *P* = 0.27) (Fig 1).

### Do bees associate hornet odor or IPA with sucrose reward?

**Fig 2:**
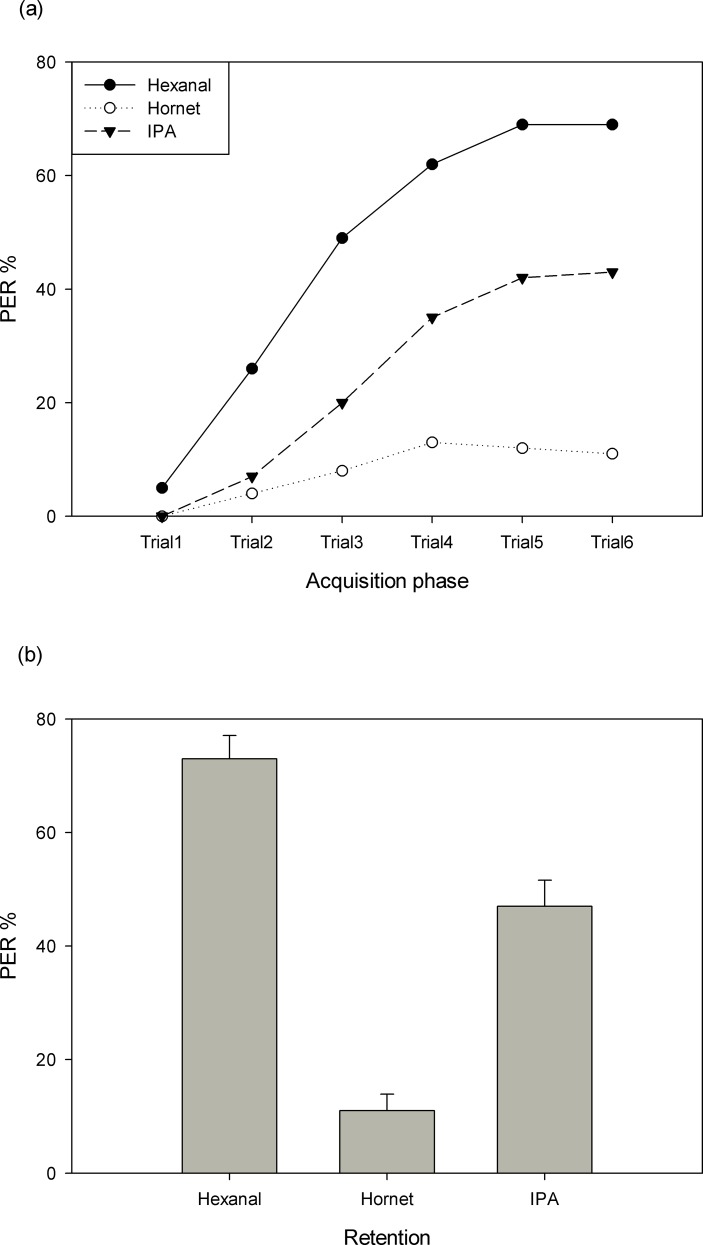
Learning and retention of hexanal, hornet odor and IPA in eastern honey bees. (a) Acquisition function of the three test groups. (b) Retention scores of the three groups as tested one hour after the last acquisition trial.

In total, 118 worker bees were tested with hornet odor, 120 worker bees with IPA and 120 bees with hexanal. The *A*. *cerana* bees showed learning scores of up to 69% when floral odors were trained. However, peak scores of only 43% were found if the alarm component IPA was trained (Fig 2A). Even lower learning scores were found when the predator odor was trained. A comparison of the three different groups showed significant differences: *A*. *cerana* learnt flower odor most, and this performance was significantly higher than for IPA or the predator odor (repeated measured ANOVA, between-subjects effects: *F*_2,355_ = 66.59, *P*<0.01). Post hoc tests also indicated similar results for each of the two groups (*P*<0.01) (Fig 2A).

Retention scores determined one hour after the last acquisition trial (Fig 2B) were highest in the hexanal group (73%), followed by the IPA group (47%), and then by the hornet odor group (11%) (*F*_2,357_ = 61.547, *P*<0.01, Fig 2).

### Does the hornet odor or the alarm odor affect the bees’ learning and memory?

**Fig 3:**
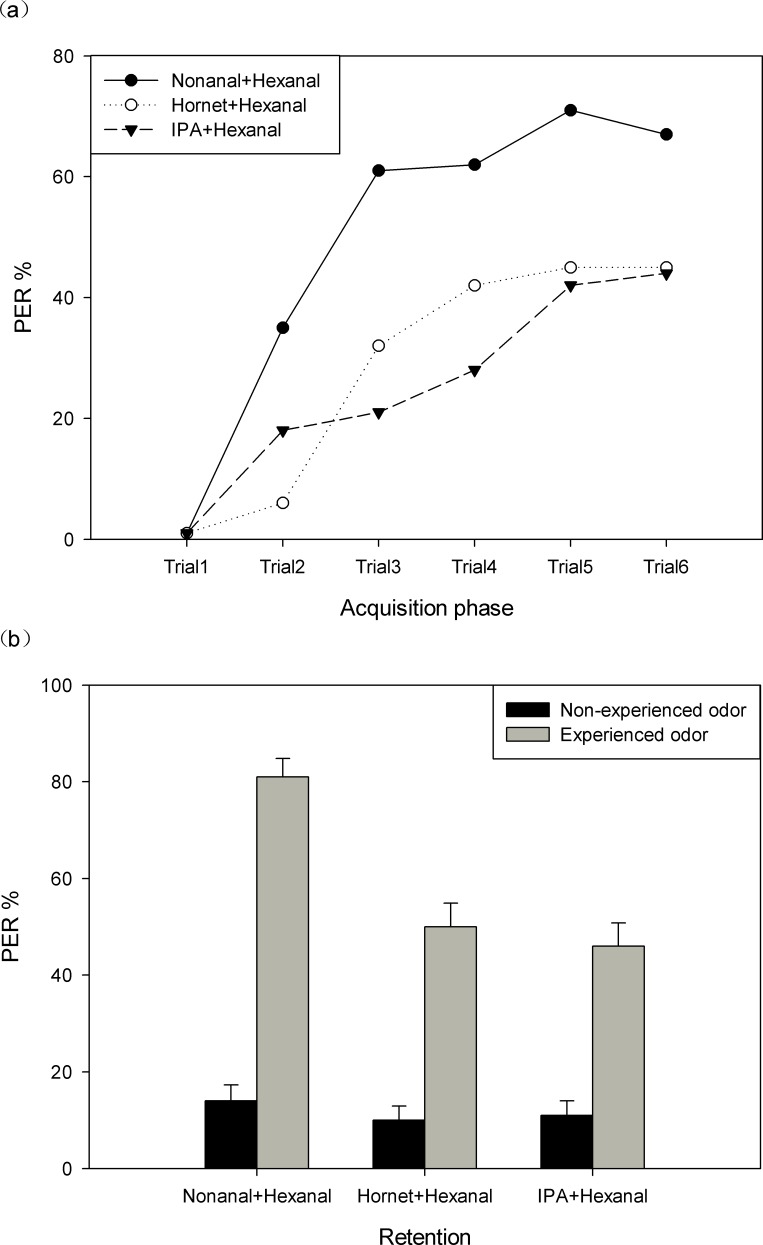
Learning and retention of three combinations of two sequential presentations of two odors: nonanal-hexanal, hornet-hexanal and IPA-hexanal. (a) Acquisition functions for the three combinations. (b) Retention scores after training to one of the three odor combinations one hour after the last acquisition trial (gray bars). In addition, the PER of three groups of naïve animals is shown for the three combinations (black bars).

Next we exposed the bees to combinations of odors (Fig 3). As expected, learning scores were highest in the nonanal-hexanal combination group (71%, *N* = 110 bees), while in the hornet odor-hexanal combination group (45%, *N* = 107 bees) and in the IPA-hexanal combination group (44%, *N* = 110 bees) they were lower and rather similar. The statistical analyses showed that acquisition in the floral odor group was significantly higher than that observed in the other two groups (repeated measures ANOVA, between-subjects effects: *F*_2,324_ = 22.75, *P*<0.01). Similar results were also found by multiple comparison analysis. In addition, those results indicated a lack of a statistically significant difference between the hornet odor group and the IPA group (*P* = 0.153, Fig 3).

This result is confirmed by the retention tests (Fig 3B, gray bars). Bees from both the hornet odor and IPA group responded with a similar low level of retention (one-way ANOVA: *F*_2,326_ = 17.801, *P*<0.01).

We introduced three other groups in these tests exposing naïve animals to the same odor combinations (Fig 3B, black bars). All three groups give the same low level of PER (hornet-hexanal combination group: 10%; IPA-hexanal combination group: 11%; nonanal-hexanal combination group with naïve animals: 14%, one way ANOVA: *F*_2,326_ = 0.336, *P* = 0.715). Paired t-tests were applied to analyze the PER between the corresponding trained and naïve groups. Bees showed strong training effects in the nonanal-hexanal combination group (*t*_109_ = 14.968, *P*<0.01) as well as in the hornet-hexanal combination group (*t*_106_ = 8.129, *P*<0.01), and the IPA-hexanal combination group (*t*_109_ = 7.738, *P*<0.01; Fig 3A and 3B).

## Discussion

In our experiments, eastern honey bees, *A*. *cerana*, can detect the odor of live hornets, and associate odor signaling a potential risk with the sucrose reward. However, acquisition scores are lower for the odor of the predator, *V*. *velutina*, and for honey bee’s alarm pheromone component, IPA, than for a floral odor (Fig 2A). Thus, compared to a floral odor, both risk-signaling odors are less well learned as predictors of reward. Furthermore, combinations of the risk-signaling odor with the floral odor reduce appetitive learning of the floral odor (Fig 3A). These data are confirmed by the retention scores of the respective test groups (Figs 2B and 3B). Predation is signaled to social bees both by odors from the predator itself and by the pheromone of attacked nest mates. Bees foraging among flower patches are subject to attack by various sit-and-wait predators (ants, bugs, mantis and spiders) [36] and other opportunist predators (hornet)[30]. Eastern honey bees have the opportunity to learn the odors of the hornet *V*. *velutina* during the summer and autumn since this wasp is endemic to Southeast Asia [37]. *V*. *velutina* have meanwhile invaded Europe causing significant threats to local apiculture of *A*. *mellifera* [28,29,38,39]. Tan and colleagues compared the different behaviors of *A*. *cerana* and *A*. *mellifera* when exposed to hornets and found that the longer evolutionary history of coexistence of *A*. *cerana* with its predators has led to more efficient defense behaviors such as more efficient shimmering behavior and heat balling [40,41]. It is thus likely that *A*. *cerana* has inherited response mechanisms to hornet odors which may not exist in *A*. *mellifera* [30].

A common defense mechanism in all *Apis* species is the release of an alarm pheromone [33,34,42] which serves to recruit more hive mates to the defense of the colony against an invader [32] or to alert mates about risky conditions at a foraging site [34]. IPA is one of main components of the alarm pheromone. IPA was first identified in *A*. *mellifera* and was shown to be an active alarm pheromone [13,43] which can be used to defend the colony [44].

Olfactory learning can be studied under laboratory conditions just as well in *A*. *cerana* as in *A*. *mellifera*, for which the appetitive PER paradigm was developed [8]. Both species associate not only floral odors but also risk-signaling odors, including the odor of the hornet predator, with reward. Not surprisingly, hornet odors and IPA are learned less well than floral odors corroborating the notion that hornet odors transmit an innate aversive component (Fig 2A). This conclusion is supported by the results of the second set of experiments showing that learning of the floral odor hexanal is reduced if hornet odors or IPA precede this floral odor (Fig 3A). The lower level of acquisition also leads to a lower level of memory as shown by the retention tests performed one hour later after the acquisition phase (Figs 2B and 3B). Similar results on the effect of IPA modulation of appetitive learning were found in *A*. *mellifera*. When bees were exposed to IPA for 30 minutes they learnt a floral odor less well than control bees not exposed to IPA [45], the main component of alarm pheromones which not only induces a stress-like response but also impairs bees learning to associate odors with sucrose reward [46]. These results correspond well with field data showing that bees reduce their foraging behavior under predation risk [34,36,47,48]. The fact that hornet odor is less well learned and remembered than IPA may indicate that bee olfactory learning is more strongly affected by direct predation risk (hornet odor) than by indirect predation risk (IPA). Our results for this pollinator-predator system (*A*. *cerana* and *V*. *velutina*) are in agreement with previous findings for *A*. *mellifera*. So why should learning of floral odor be lower when bees are exposed to risk-signaling conditions? This question may be put into the context of how bees should react to direct and indirect predation risk. Optimal defensive theory and optimal foraging theory suggest that bees need to maximize their foraging efficiency and minimize risk posed by predators. If bees were to stop foraging altogether in response to an overall high risk, the colony might suffer. Thus bees may need to assess the current risk and behave accordingly. Higher risk may be signaled by the odor of the predator, lower risk by the alarm pheromone since the predator is not in the immediate surroundings, and the alarm pheromone alerts the bee but may not trigger an immediate response. Furthermore, floral odors may be experienced in sequence with or without risk-signaling odors. Again hornet odors would signal a higher risk than alarm pheromone. Better learning of floral odors without risk-signaling odors or only in combination with alarm odors would lead to a preference for these signals over hornet-signaling odors.

Similar conditions may apply at the hive entrance when the whole colony needs to be protected. Ono et al. [49] reported that the Japanese honey bee *A*. *cerana japonica* can detect the hornet-marking pheromone (pheromones from the van der Vecht glands of hornet, which were used to detect the prey locations): when one defending bee captured a hornet, more than 500 additional worker bees were soon recruited via IPA (isoamyl acetate in Ono et al., 1995) to form a heat ball against predator invasion. A similar phenomenon was found in *A*. *cerana cerana*: when a hornet hovered around the entrance of a bee hive, guard bees were able to detect an increasing risk and the closer the hornet came, the more bees were recruited to shimmer [31]. Even the naïve *A*. *cerana* bee evolved to shaking their body to repel the predator at an early age [30].

Reduced learning and memory of a floral odor in the context of a risk-signaling odor could reflect an innate preparedness of the animal to avoid the appetitive signal or it could reflect a conflict between two opposing behaviors. These behaviors would be an appetitive approach in response to the learned floral odor and innate retraction or attack of the risk-signaling odor. The latter behaviors could lead to aversive learning and/or to blocking of appetitive learning. Our results show that learning of floral odors is not blocked under risk conditions, and no indication for aversive or aggressive responses is seen (e.g. sting extension). It appears, therefore, that two opposing behaviors may compete leading to a lower level of appetitive responsiveness to the learned floral odor. Conditioning of the sting extension response (SER) [50] may help to unravel the acting conditions. SER conditioning would not lead to such a competition of behaviors. Therefore, one would expect that risk-signaling odors are better learned and that floral odors produce competing behaviors.
